# Supplementary material for: Less Severe Polymicrobial Sepsis in Conditional mgmt-Deleted Mice Using LysM-Cre System, Impacts of DNA Methylation and MGMT Inhibitor in Sepsis
Source: Int J Mol Sci. 2023 Jun 15;24(12):10175. doi: 10.3390/ijms241210175 (PMC10299382; doi:10.3390/ijms241210175)
Supplement: Supplementary file 1 [file ijms-24-10175-s001.zip › ijms-2421894-supplementary.pdf]

Table S1. The genes responsible for elevated proteins in LPS-activated WT macrophages

Pasted	Symbol	C*	P**	Description
LMBRD1	Lmbrd1	1	25	LMBR1 domain containing 1 [Source:MGI Symbol;Acc:MGI:1915671]
STAT1	Stat1	1	52	signal transducer and activator of transcription 1 [Source:MGI Symbol;Acc:MGI:103063]
CASP8	Casp8	1	59	caspase 8 [Source:MGI Symbol;Acc:MGI:1261423]
AGFG1	Agfg1	1	83	ArfGAP with FG repeats 1 [Source:MGI Symbol;Acc:MGI:1333754]
TRIP12	Trip12	1	85	thyroid hormone receptor interactor 12 [Source:MGI Symbol;Acc:MGI:1309481]
MNDA	Ifi211	1	174	interferon activated gene 211 [Source:MGI Symbol;Acc:MGI:3041120]
APBB1IP	Apbb1ip	2	23	amyloid beta (A4) precursor protein-binding, family B, member 1 interacting protein [Source:MGI Symbol;Acc:MGI:1861354]
IL1RN	Il1rn	2	24	interleukin 1 receptor antagonist [Source:MGI Symbol;Acc:MGI:96547]
EDF1	Edf1	2	25	endothelial differentiation-related factor 1 [Source:MGI Symbol;Acc:MGI:1891227]
SEC16A	Sec16a	2	26	SEC16 homolog A, endoplasmic reticulum export factor [Source:MGI Symbol;Acc:MGI:2139207]
TBC1D13	Tbc1d13	2	30	TBC1 domain family, member 13 [Source:MGI Symbol;Acc:MGI:2385326]
ASS1	Ass1	2	31	argininosuccinate synthetase 1 [Source:MGI Symbol;Acc:MGI:88090]
NMI	Nmi	2	52	N-myc (and STAT) interactor [Source:MGI Symbol;Acc:MGI:1928368]
STAM2	Stam2	2	53	signal transducing adaptor molecule (SH3 domain and ITAM motif) 2 [Source:MGI Symbol;Acc:MGI:1929100]
GPD2	Gpd2	2	57	glycerol phosphate dehydrogenase 2, mitochondrial [Source:MGI Symbol;Acc:MGI:99778]
SSB	Ssb	2	70	Sjogren syndrome antigen B [Source:MGI Symbol;Acc:MGI:98423]
DYNC1I2	Dync1i2	2	71	dynein cytoplasmic 1 intermediate chain 2 [Source:MGI Symbol;Acc:MGI:107750]
DNAJC10	Dnajc10	2	80	DnaJ heat shock protein family (Hsp40) member C10 [Source:MGI Symbol;Acc:MGI:1914111]
CAPRIN1	Caprin1	2	104	cell cycle associated protein 1 [Source:MGI Symbol;Acc:MGI:1858234]
B2M	B2m	2	122	beta-2 microglobulin [Source:MGI Symbol;Acc:MGI:88127]
IL1B	Il1b	2	129	interleukin 1 beta [Source:MGI Symbol;Acc:MGI:96543]
CHMP4B	Chmp4b	2	154	charged multivesicular body protein 4B [Source:MGI Symbol;Acc:MGI:1922858]

AAR2	Aar2	2	156	AAR2 splicing factor homolog [Source:MGI Symbol;Acc:MGI:1915545]
CSE1L	Cse1l	2	167	chromosome segregation 1-like (S. cerevisiae) [Source:MGI Symbol;Acc:MGI:1339951]
CEBPB	Cebpb	2	168	CCAAT/enhancer binding protein (C/EBP), beta [Source:MGI Symbol;Acc:MGI:88373]
CA13	Car13	3	15	carbonic anhydrase 13 [Source:MGI Symbol;Acc:MGI:1931322]
MCL1	Mcl1	3	96	myeloid cell leukemia sequence 1 [Source:MGI Symbol;Acc:MGI:101769]
FCGR1	Fcgr1	3	96	Fc receptor, IgG, high affinity I [Source:MGI Symbol;Acc:MGI:95498]
MOV10	Mov10	3	105	Mov10 RISC complex RNA helicase [Source:MGI Symbol;Acc:MGI:97054]
GNAI3	Gnai3	3	108	guanine nucleotide binding protein (G protein), alpha inhibiting 3 [Source:MGI Symbol;Acc:MGI:95773]
GBP5	Gbp5	3	142	guanylate binding protein 5 [Source:MGI Symbol;Acc:MGI:2429943]
GBP7	Gbp7	3	142	guanylate binding protein 7 [Source:MGI Symbol;Acc:MGI:2444421]
GBP2	Gbp2	3	142	guanylate binding protein 2 [Source:MGI Symbol;Acc:MGI:102772]
BCL10	Bcl10	3	146	B cell leukemia/lymphoma 10 [Source:MGI Symbol;Acc:MGI:1337994]
DDX58	Ddx58	4	40	DEAD/H box helicase 58 [Source:MGI Symbol;Acc:MGI:2442858]
DNAJA1	Dnaja1	4	41	DnaJ heat shock protein family (Hsp40) member A1 [Source:MGI Symbol;Acc:MGI:1270129]
UBR4	Ubr4	4	139	ubiquitin protein ligase E3 component n-recognin 4 [Source:MGI Symbol;Acc:MGI:1916366]
PRXL2B	Prxl2b	4	155	peroxiredoxin like 2B [Source:MGI Symbol;Acc:MGI:1913719]
NADK	Nadk	4	156	NAD kinase [Source:MGI Symbol;Acc:MGI:2183149]
ABCF2	Abcf2	5	25	ATP-binding cassette, sub-family F (GCN20), member 2 [Source:MGI Symbol;Acc:MGI:1351657]
MRFAP1	Mrfap1	5	37	Morf4 family associated protein 1 [Source:MGI Symbol;Acc:MGI:1914818]
DHX15	Dhx15	5	52	DEAH (Asp-Glu-Ala-His) box polypeptide 15 [Source:MGI Symbol;Acc:MGI:1099786]
KCTD8	Kctd8	5	69	potassium channel tetramerisation domain containing 8 [Source:MGI Symbol;Acc:MGI:2443804]
DCK	Dck	5	89	deoxycytidine kinase [Source:MGI Symbol;Acc:MGI:102726]
GBP6	Gbp6	5	105	guanylate binding protein 6 [Source:MGI Symbol;Acc:MGI:2140937]
RPL5	Rpl5	5	108	ribosomal protein L5 [Source:MGI Symbol;Acc:MGI:102854]
GOLGA3	Golga3	5	110	golgi autoantigen, golgin subfamily a, 3 [Source:MGI Symbol;Acc:MGI:96958]
TPST2	Tpst2	5	112	protein-tyrosine sulfotransferase 2 [Source:MGI Symbol;Acc:MGI:1309516]
OASL1	Oasl1	5	115	2'-5' oligoadenylate synthetase-like 1 [Source:MGI Symbol;Acc:MGI:2180849]

OAS3	Oas3	5	121	2'-5' oligoadenylate synthetase 3 [Source:MGI Symbol;Acc:MGI:2180850]
OAS1A	Oas1a	5	121	2'-5' oligoadenylate synthetase 1A [Source:MGI Symbol;Acc:MGI:2180860]
PLOD3	Plod3	5	137	procollagen-lysine, 2-oxoglutarate 5-dioxygenase 3 [Source:MGI Symbol;Acc:MGI:1347008]
COPS6	Cops6	5	138	COP9 signalosome subunit 6 [Source:MGI Symbol;Acc:MGI:1349439]
AKR1B1	Akr1b3	6	34	aldo-keto reductase family 1, member B3 (aldose reductase) [Source:MGI Symbol;Acc:MGI:1353494]
MTPN	Mtpn	6	35	myotrophin [Source:MGI Symbol;Acc:MGI:99445]
TBXAS1	Tbxas1	6	39	thromboxane A synthase 1, platelet [Source:MGI Symbol;Acc:MGI:98497]
NT5C3A	Nt5c3	6	57	5'-nucleotidase, cytosolic III [Source:MGI Symbol;Acc:MGI:1927186]
KCMF1	Kcmf1	6	73	potassium channel modulatory factor 1 [Source:MGI Symbol;Acc:MGI:1921537]
HK2	Hk2	6	83	hexokinase 2 [Source:MGI Symbol;Acc:MGI:1315197]
COPG1	Copg1	6	88	coatomer protein complex, subunit gamma 1 [Source:MGI Symbol;Acc:MGI:1858696]
RASSF4	Rassf4	6	117	Ras association (RalGDS/AF-6) domain family member 4 [Source:MGI Symbol;Acc:MGI:2386853]
BID	Bid	6	121	BH3 interacting domain death agonist [Source:MGI Symbol;Acc:MGI:108093]
USP18	Usp18	6	121	ubiquitin specific peptidase 18 [Source:MGI Symbol;Acc:MGI:1344364]
ARHGDIB	Arhgdib	6	137	Rho, GDP dissociation inhibitor (GDI) beta [Source:MGI Symbol;Acc:MGI:101940]
NECTIN2	Nectin2	7	19	nectin cell adhesion molecule 2 [Source:MGI Symbol;Acc:MGI:97822]
GPI	Gpi1	7	34	glucose-6-phosphate isomerase 1 [Source:MGI Symbol;Acc:MGI:95797]
GYS1	Gys1	7	45	glycogen synthase 1, muscle [Source:MGI Symbol;Acc:MGI:101805]
SAA3	Saa3	7	46	serum amyloid A 3 [Source:MGI Symbol;Acc:MGI:98223]
MESD	Mesd	7	84	mesoderm development LRP chaperone [Source:MGI Symbol;Acc:MGI:1891421]
CLNS1A	Clns1a	7	97	chloride channel, nucleotide-sensitive, 1A [Source:MGI Symbol;Acc:MGI:109638]
TRIM21	Trim21	7	102	tripartite motif-containing 21 [Source:MGI Symbol;Acc:MGI:106657]
TRIM30A	Trim30a	7	104	tripartite motif-containing 30A [Source:MGI Symbol;Acc:MGI:98178]
ITGAL	Itgal	7	127	integrin alpha L [Source:MGI Symbol;Acc:MGI:96606]
PSMD13	Psmd13	7	140	proteasome (prosome, macropain) 26S subunit, non-ATPase, 13 [Source:MGI Symbol;Acc:MGI:1345192]
MSR1	Msr1	8	40	macrophage scavenger receptor 1 [Source:MGI Symbol;Acc:MGI:98257]
TMA16	Tma16	8	67	translation machinery associated 16 [Source:MGI Symbol;Acc:MGI:1913532]

LRRC25	Lrrc25	8	71	leucine rich repeat containing 25 [Source:MGI Symbol;Acc:MGI:2445284]
CCDC124	Ccdc124	8	71	coiled-coil domain containing 124 [Source:MGI Symbol;Acc:MGI:1916403]
BST2	Bst2	8	72	bone marrow stromal cell antigen 2 [Source:MGI Symbol;Acc:MGI:1916800]
HMOX1	Hmox1	8	76	heme oxygenase 1 [Source:MGI Symbol;Acc:MGI:96163]
TRMT1	Trmt1	8	85	tRNA methyltransferase 1 [Source:MGI Symbol;Acc:MGI:1289155]
LPCAT2	Lpcat2	8	94	lysophosphatidylcholine acyltransferase 2 [Source:MGI Symbol;Acc:MGI:3606214]
PSME3IP1	Psme3ip1	8	95	proteasome activator subunit 3 interacting protein 1 [Source:MGI Symbol;Acc:MGI:1919637]
CIAO2B	Ciao2b	8	105	cytosolic iron-sulfur assembly component 2B [Source:MGI Symbol;Acc:MGI:1915773]
PSMD7	Psmd7	8	108	proteasome (prosome, macropain) 26S subunit, non-ATPase, 7 [Source:MGI Symbol;Acc:MGI:1351511]
EIF3G	Eif3g	9	21	eukaryotic translation initiation factor 3, subunit G [Source:MGI Symbol;Acc:MGI:1858258]
ICAM1	Icam1	9	21	intercellular adhesion molecule 1 [Source:MGI Symbol;Acc:MGI:96392]
ILF3	Ilf3	9	21	interleukin enhancer binding factor 3 [Source:MGI Symbol;Acc:MGI:1339973]
CBL	Cbl	9	44	Casitas B-lineage lymphoma [Source:MGI Symbol;Acc:MGI:88279]
RPS25	Rps25	9	44	ribosomal protein S25 [Source:MGI Symbol;Acc:MGI:1922867]
ARCN1	Arcn1	9	45	archain 1 [Source:MGI Symbol;Acc:MGI:2387591]
PML	Pml	9	58	promyelocytic leukemia [Source:MGI Symbol;Acc:MGI:104662]
RPLP1	Rplp1	9	62	ribosomal protein, large, P1 [Source:MGI Symbol;Acc:MGI:1927099]
ANP32A	Anp32a	9	62	acidic (leucine-rich) nuclear phosphoprotein 32 family, member A [Source:MGI Symbol;Acc:MGI:108447]
MAP2K1	Map2k1	9	64	mitogen-activated protein kinase kinase 1 [Source:MGI Symbol;Acc:MGI:1346866]
COPB2	Copb2	9	98	coatomer protein complex, subunit beta 2 (beta prime) [Source:MGI Symbol;Acc:MGI:1354962]
MANF	Manf	9	107	mesencephalic astrocyte-derived neurotrophic factor [Source:MGI Symbol;Acc:MGI:1922090]
MAP4	Map4	9	110	microtubule-associated protein 4 [Source:MGI Symbol;Acc:MGI:97178]
CLASP2	Clasp2	9	114	CLIP associating protein 2 [Source:MGI Symbol;Acc:MGI:1923749]
LZTFL1	Lztfl1	9	124	leucine zipper transcription factor-like 1 [Source:MGI Symbol;Acc:MGI:1934860]
VPS26A	Vps26a	10	62	VPS26 retromer complex component A [Source:MGI Symbol;Acc:MGI:1353654]
CCDC6	Ccdc6	10	70	coiled-coil domain containing 6 [Source:MGI Symbol;Acc:MGI:1923801]

PFKL	Pfkl	10	78	phosphofructokinase, liver, B-type [Source:MGI Symbol;Acc:MGI:97547]
BSG	Bsg	10	80	basigin [Source:MGI Symbol;Acc:MGI:88208]
RPS15	Rps15	10	80	ribosomal protein S15 [Source:MGI Symbol;Acc:MGI:98117]
CPSF6	Cpsf6	10	117	cleavage and polyadenylation specific factor 6 [Source:MGI Symbol;Acc:MGI:1913948]
STAT2	Stat2	10	128	signal transducer and activator of transcription 2 [Source:MGI Symbol;Acc:MGI:103039]
DBNL	Dbnl	11	6	drebrin-like [Source:MGI Symbol;Acc:MGI:700006]
PLEK	Plek	11	17	pleckstrin [Source:MGI Symbol;Acc:MGI:1860485]
RAB1A	Rab1a	11	20	RAB1A, member RAS oncogene family [Source:MGI Symbol;Acc:MGI:97842]
CCT4	Cct4	11	23	chaperonin containing Tcp1, subunit 4 (delta) [Source:MGI Symbol;Acc:MGI:104689]
REL	Rel	11	24	reticuloendotheliosis oncogene [Source:MGI Symbol;Acc:MGI:97897]
LCP2	Lcp2	11	34	lymphocyte cytosolic protein 2 [Source:MGI Symbol;Acc:MGI:1321402]
PSME2	Psme2b	11	49	protease (prosome, macropain) activator subunit 2B [Source:MGI Symbol;Acc:MGI:1341073]
SQSTM1	Sqstm1	11	50	sequestosome 1 [Source:MGI Symbol;Acc:MGI:107931]
HSPA4	Hspa4	11	53	heat shock protein 4 [Source:MGI Symbol;Acc:MGI:1342292]
ZZEF1	Zzef1	11	73	zinc finger, ZZ-type with EF hand domain 1 [Source:MGI Symbol;Acc:MGI:2444286]
PAFAH1B1	Pafah1b1	11	75	platelet-activating factor acetylhydrolase, isoform 1b, subunit 1 [Source:MGI Symbol;Acc:MGI:109520]
BLMH	Blmh	11	77	bleomycin hydrolase [Source:MGI Symbol;Acc:MGI:1345186]
NUFIP2	Nufip2	11	78	nuclear fragile X mental retardation protein interacting protein 2 [Source:MGI Symbol;Acc:MGI:1915814]
RPL23A	Rpl23a	11	78	ribosomal protein L23A [Source:MGI Symbol;Acc:MGI:3040672]
LGALS9	Lgals9	11	79	lectin, galactose binding, soluble 9 [Source:MGI Symbol;Acc:MGI:109496]
CRLF3	Crlf3	11	80	cytokine receptor-like factor 3 [Source:MGI Symbol;Acc:MGI:1860086]
ZNF207	Zfp207	11	80	zinc finger protein 207 [Source:MGI Symbol;Acc:MGI:1340045]
PSMD11	Psmd11	11	80	proteasome (prosome, macropain) 26S subunit, non-ATPase, 11 [Source:MGI Symbol;Acc:MGI:1916327]
PSMD3	Psmd3	11	99	proteasome (prosome, macropain) 26S subunit, non-ATPase, 3 [Source:MGI Symbol;Acc:MGI:98858]
EIF1	Eif1	11	100	eukaryotic translation initiation factor 1 [Source:MGI Symbol;Acc:MGI:105125]
DHX58	Dhx58	11	101	DEXH (Asp-Glu-X-His) box polypeptide 58 [Source:MGI Symbol;Acc:MGI:1931560]

LGALS3BP	Lgals3bp	11	118	lectin, galactoside-binding, soluble, 3 binding protein [Source:MGI Symbol;Acc:MGI:99554]
NPLOC4	Nploc4	11	120	NPL4 homolog, ubiquitin recognition factor [Source:MGI Symbol;Acc:MGI:2679787]
CYRIA	Cyria	12	12	CYFIP related Rac1 interactor A [Source:MGI Symbol;Acc:MGI:1261783]
NAMPT	Nampt	12	33	nicotinamide phosphoribosyltransferase [Source:MGI Symbol;Acc:MGI:1929865]
DDX24	Ddx24	12	103	DEAD box helicase 24 [Source:MGI Symbol;Acc:MGI:1351337]
EIF5	Eif5	12	112	eukaryotic translation initiation factor 5 [Source:MGI Symbol;Acc:MGI:95309]
HK3	Hk3	13	55	hexokinase 3 [Source:MGI Symbol;Acc:MGI:2670962]
CAST	Cast	13	75	calpastatin [Source:MGI Symbol;Acc:MGI:1098236]
PSMD6	Psmd6	14	8	proteasome (prosome, macropain) 26S subunit, non-ATPase, 6 [Source:MGI Symbol;Acc:MGI:1913663]
FLNB	Flnb	14	15	filamin, beta [Source:MGI Symbol;Acc:MGI:2446089]
RTRAF	Rtraf	14	20	RNA transcription, translation and transport factor [Source:MGI Symbol;Acc:MGI:1915295]
RPS24	Rps24	14	25	ribosomal protein S24 [Source:MGI Symbol;Acc:MGI:98147]
PNP	Pnp	14	51	purine-nucleoside phosphorylase [Source:MGI Symbol;Acc:MGI:97365]
ACIN1	Acin1	14	55	apoptotic chromatin condensation inducer 1 [Source:MGI Symbol;Acc:MGI:1891824]
PSME1	Psme1	14	56	proteasome (prosome, macropain) activator subunit 1 (PA28 alpha) [Source:MGI Symbol;Acc:MGI:1096367]
FNDC3A	Fndc3a	14	73	fibronectin type III domain containing 3A [Source:MGI Symbol;Acc:MGI:1196463]
ACOD1	Acod1	14	103	aconitate decarboxylase 1 [Source:MGI Symbol;Acc:MGI:103206]
GSDMD	Gsdmd	15	76	gasdermin D [Source:MGI Symbol;Acc:MGI:1916396]
NCF4	Ncf4	15	78	neutrophil cytosolic factor 4 [Source:MGI Symbol;Acc:MGI:109186]
BIN2	Bin2	15	101	bridging integrator 2 [Source:MGI Symbol;Acc:MGI:3611448]
ITGA5	Itga5	15	103	integrin alpha 5 (fibronectin receptor alpha) [Source:MGI Symbol;Acc:MGI:96604]
NCKAP1L	Nckap1l	15	103	NCK associated protein 1 like [Source:MGI Symbol;Acc:MGI:1926063]
GSPT1	Gspt1	16	11	G1 to S phase transition 1 [Source:MGI Symbol;Acc:MGI:1316728]
DNAJB11	Dnajb11	16	23	DnaJ heat shock protein family (Hsp40) member B11 [Source:MGI Symbol;Acc:MGI:1915088]
HCLS1	Hcls1	16	37	hematopoietic cell specific Lyn substrate 1 [Source:MGI Symbol;Acc:MGI:104568]
COX17	Cox17	16	38	cytochrome c oxidase assembly protein 17, copper chaperone [Source:MGI Symbol;Acc:MGI:1333806]

FILIP1L	Filip1l	16	57	filamin A interacting protein 1-like [Source:MGI Symbol;Acc:MGI:1925999]
CLCN7	Clcn7	17	25	chloride channel, voltage-sensitive 7 [Source:MGI Symbol;Acc:MGI:1347048]
RPL10A	Rpl10a	17	29	ribosomal protein L10A [Source:MGI Symbol;Acc:MGI:1343877]
RPS28	Rps28	17	34	ribosomal protein S28 [Source:MGI Symbol;Acc:MGI:1859516]
H2-K1	H2-K1	17	34	histocompatibility 2, K1, K region [Source:MGI Symbol;Acc:MGI:95904]
TAP1	Tap1	17	34	transporter 1, ATP-binding cassette, sub-family B (MDR/TAP) [Source:MGI Symbol;Acc:MGI:98483]
TAP2	Tap2	17	34	transporter 2, ATP-binding cassette, sub-family B (MDR/TAP) [Source:MGI Symbol;Acc:MGI:98484]
HSP90AB1	Hsp90ab1	17	46	heat shock protein 90 alpha (cytosolic), class B member 1 [Source:MGI Symbol;Acc:MGI:96247]
XPO5	Xpo5	17	47	exportin 5 [Source:MGI Symbol;Acc:MGI:1913789]
GTF2F1	Gtf2f1	17	57	general transcription factor IIF, polypeptide 1 [Source:MGI Symbol;Acc:MGI:1923848]
C3	C3	17	58	complement component 3 [Source:MGI Symbol;Acc:MGI:88227]
EIF2AK2	Eif2ak2	17	79	eukaryotic translation initiation factor 2-alpha kinase 2 [Source:MGI Symbol;Acc:MGI:1353449]
MAPRE2	Mapre2	18	24	microtubule-associated protein, RP/EB family, member 2 [Source:MGI Symbol;Acc:MGI:106271]
PFDN1	Pfdn1	18	37	prefoldin 1 [Source:MGI Symbol;Acc:MGI:1914449]
EIF1A	Eif1a	18	47	eukaryotic translation initiation factor 1A [Source:MGI Symbol;Acc:MGI:95298]
MALT1	Malt1	18	66	MALT1 paracaspase [Source:MGI Symbol;Acc:MGI:2445027]
GSTP1	Gstp1	19	4	glutathione S-transferase, pi 1 [Source:MGI Symbol;Acc:MGI:95865]
SSH3	Ssh3	19	4	slingshot protein phosphatase 3 [Source:MGI Symbol;Acc:MGI:2683546]
FRMD8	Frmd8	19	6	FERM domain containing 8 [Source:MGI Symbol;Acc:MGI:1914707]
ZFPL1	Zfpl1	19	6	zinc finger like protein 1 [Source:MGI Symbol;Acc:MGI:1891017]
EHD1	Ehd1	19	6	EH-domain containing 1 [Source:MGI Symbol;Acc:MGI:1341878]
PRDX5	Prdx5	19	7	peroxiredoxin 5 [Source:MGI Symbol;Acc:MGI:1859821]
MS4A6B	Ms4a6b	19	11	membrane-spanning 4-domains, subfamily A, member 6B [Source:MGI Symbol;Acc:MGI:1917024]
OSBP	Osbp	19	12	oxysterol binding protein [Source:MGI Symbol;Acc:MGI:97447]
DOCK8	Dock8	19	25	dedicator of cytokinesis 8 [Source:MGI Symbol;Acc:MGI:1921396]

IFIT2	Ifit2	19	35	interferon-induced protein with tetratricopeptide repeats 2 [Source:MGI Symbol;Acc:MGI:99449]
IFIT3	Ifit3	19	35	interferon-induced protein with tetratricopeptide repeats 3 [Source:MGI Symbol;Acc:MGI:1101055]
IFIT1	Ifit1	19	35	interferon-induced protein with tetratricopeptide repeats 1 [Source:MGI Symbol;Acc:MGI:99450]
NFKB2	Nfkb2	19	46	nuclear factor of kappa light polypeptide gene enhancer in B cells 2, p49/p100 [Source:MGI Symbol;Acc:MGI:1099800]
VTI1A	Vti1a	19	55	vesicle transport through interaction with t-SNAREs 1A [Source:MGI Symbol;Acc:MGI:1855699]
IGBP1	Igbp1	X	100	immunoglobulin (CD79A) binding protein 1 [Source:MGI Symbol;Acc:MGI:1346500]
PSMD10	Psmd10	X	140	proteasome (prosome, macropain) 26S subunit, non-ATPase, 10 [Source:MGI Symbol;Acc:MGI:1858898]

* Chromosome; **, Position (Mbp)

Table S2. The genes responsible for decreased proteins in LPS-activated WT macrophages

Pasted	Symbol	C*	P**	Description
RPL7	Rpl7	1	16	ribosomal protein L7 [Source:MGI Symbol;Acc:MGI:98073]
COX5B	Cox5b	1	37	cytochrome c oxidase subunit 5B [Source:MGI Symbol;Acc:MGI:88475]
HSPD1	Hspd1	1	55	heat shock protein 1 (chaperonin) [Source:MGI Symbol;Acc:MGI:96242]
DES	Des	1	75	desmin [Source:MGI Symbol;Acc:MGI:94885]
NDUFA10	Ndufa10	1	92	NADH:ubiquinone oxidoreductase subunit A10 [Source:MGI Symbol;Acc:MGI:1914523]
CDK18	Cdk18	1	132	cyclin-dependent kinase 18 [Source:MGI Symbol;Acc:MGI:97518]
SNRPE	Snrpe	1	134	small nuclear ribonucleoprotein E [Source:MGI Symbol;Acc:MGI:98346]
GLUL	Glul	1	154	glutamate-ammonia ligase (glutamine synthetase) [Source:MGI Symbol;Acc:MGI:95739]
FH	Fh1	1	175	fumarate hydratase 1 [Source:MGI Symbol;Acc:MGI:95530]
CAT	Cat	2	103	catalase [Source:MGI Symbol;Acc:MGI:88271]
IVD	Ivd	2	119	isovaleryl coenzyme A dehydrogenase [Source:MGI Symbol;Acc:MGI:1929242]
GALK2	Galk2	2	126	galactokinase 2 [Source:MGI Symbol;Acc:MGI:1917226]
CST3	Cst3	2	149	cystatin C [Source:MGI Symbol;Acc:MGI:102519]
EPB41L1	Epb41l1	2	156	erythrocyte membrane protein band 4.1 like 1 [Source:MGI Symbol;Acc:MGI:103010]

RAE1	Rae1	2	173	ribonucleic acid export 1 [Source:MGI Symbol;Acc:MGI:1913929]
RNF13	Rnf13	3	58	ring finger protein 13 [Source:MGI Symbol;Acc:MGI:1346341]
PLEKHO1	Plekho1	3	96	pleckstrin homology domain containing, family O member 1 [Source:MGI Symbol;Acc:MGI:1914470]
CHIL3	Chil3	3	106	chitinase-like 3 [Source:MGI Symbol;Acc:MGI:1330860]
GCLM	Gclm	3	122	glutamate-cysteine ligase, modifier subunit [Source:MGI Symbol;Acc:MGI:104995]
CAMK2D	Camk2d	3	126	calcium/calmodulin-dependent protein kinase II, delta [Source:MGI Symbol;Acc:MGI:1341265]
RPL34	Rpl34	3	131	ribosomal protein L34 [Source:MGI Symbol;Acc:MGI:1915686]
DECR1	Decr1	4	16	2,4-dienoyl CoA reductase 1, mitochondrial [Source:MGI Symbol;Acc:MGI:1914710]
STOML2	Stoml2	4	43	stomatin (Epb7.2)-like 2 [Source:MGI Symbol;Acc:MGI:1913842]
HINT2	Hint2	4	44	histidine triad nucleotide binding protein 2 [Source:MGI Symbol;Acc:MGI:1916167]
PRDX1	Prdx1	4	117	peroxiredoxin 1 [Source:MGI Symbol;Acc:MGI:99523]
HMGCL	Hmgcl	4	136	3-hydroxy-3-methylglutaryl-Coenzyme A lyase [Source:MGI Symbol;Acc:MGI:96158]
C1QC	C1qc	4	137	complement component 1, q subcomponent, C chain [Source:MGI Symbol;Acc:MGI:88225]
CD36	Cd36	5	18	CD36 molecule [Source:MGI Symbol;Acc:MGI:107899]
HADHA	Hadha	5	30	hydroxyacyl-CoA dehydrogenase trifunctional multienzyme complex subunit alpha [Source:MGI Symbol;Acc:MGI:2135593]
HADHB	Hadhb	5	30	hydroxyacyl-CoA dehydrogenase trifunctional multienzyme complex subunit beta [Source:MGI Symbol;Acc:MGI:2136381]
MAN2B2	Man2b2	5	37	mannosidase 2, alpha B2 [Source:MGI Symbol;Acc:MGI:1195262]
COMMD8	Commd8	5	72	COMM domain containing 8 [Source:MGI Symbol;Acc:MGI:1343485]
PAICS	Paics	5	77	phosphoribosylaminoimidazole carboxylase, phosphoribosylaminoribosylaminoimidazole, succinocarboxamide synthetase [Source:MGI Symbol;Acc:MGI:1914304]
STAP1	Stap1	5	86	signal transducing adaptor family member 1 [Source:MGI Symbol;Acc:MGI:1926193]
ANXA3	Anxa3	5	97	annexin A3 [Source:MGI Symbol;Acc:MGI:1201378]
IDUA	Idua	5	109	iduronidase, alpha-L [Source:MGI Symbol;Acc:MGI:96418]
HSCB	Hscb	5	111	HscB iron-sulfur cluster co-chaperone [Source:MGI Symbol;Acc:MGI:2141135]
ALDH2	Aldh2	5	122	aldehyde dehydrogenase 2, mitochondrial [Source:MGI Symbol;Acc:MGI:99600]
NDUFA4	Ndufa4	6	12	Ndufa4, mitochondrial complex associated [Source:MGI Symbol;Acc:MGI:107686]
AKR1B8	Akr1b8	6	34	aldo-keto reductase family 1, member B8 [Source:MGI Symbol;Acc:MGI:107673]

ATP6V1E1	Atp6v1e1	6	121	ATPase, H+ transporting, lysosomal V1 subunit E1 [Source:MGI Symbol;Acc:MGI:894326]
PPFIBP1	Ppfibp1	6	147	PTPRF interacting protein, binding protein 1 (liprin beta 1) [Source:MGI Symbol;Acc:MGI:1914783]
SAE1	Sae1	7	16	SUMO1 activating enzyme subunit 1 [Source:MGI Symbol;Acc:MGI:1929264]
CKM	Ckm	7	19	creatine kinase, muscle [Source:MGI Symbol;Acc:MGI:88413]
BLOC1S3	Bloc1s3	7	19	biogenesis of lysosomal organelles complex-1, subunit 3 [Source:MGI Symbol;Acc:MGI:2678952]
APOE	Apoe	7	19	apolipoprotein E [Source:MGI Symbol;Acc:MGI:88057]
BLVRB	Blvrb	7	27	biliverdin reductase B (flavin reductase (NADPH)) [Source:MGI Symbol;Acc:MGI:2385271]
PLD3	Pld3	7	27	phospholipase D family, member 3 [Source:MGI Symbol;Acc:MGI:1333782]
ETFB	Etfb	7	43	electron transferring flavoprotein, beta polypeptide [Source:MGI Symbol;Acc:MGI:106098]
NUCB1	Nucb1	7	45	nucleobindin 1 [Source:MGI Symbol;Acc:MGI:97388]
BCAT2	Bcat2	7	45	branched chain aminotransferase 2, mitochondrial [Source:MGI Symbol;Acc:MGI:1276534]
IDH2	Idh2	7	80	isocitrate dehydrogenase 2 (NADP+), mitochondrial [Source:MGI Symbol;Acc:MGI:96414]
FOLR2	Folr2	7	101	folate receptor 2 (fetal) [Source:MGI Symbol;Acc:MGI:95569]
LAMTOR1	Lamtor1	7	102	late endosomal/lysosomal adaptor, MAPK and MTOR activator 1 [Source:MGI Symbol;Acc:MGI:1913758]
TUFM	Tufm	7	126	Tu translation elongation factor, mitochondrial [Source:MGI Symbol;Acc:MGI:1923686]
PYCARD	Pycard	7	128	PYD and CARD domain containing [Source:MGI Symbol;Acc:MGI:1931465]
ADAM8	Adam8	7	140	a disintegrin and metallopeptidase domain 8 [Source:MGI Symbol;Acc:MGI:107825]
GSR	Gsr	8	34	glutathione reductase [Source:MGI Symbol;Acc:MGI:95804]
LPL	Lpl	8	69	lipoprotein lipase [Source:MGI Symbol;Acc:MGI:96820]
PGLS	Pgls	8	72	6-phosphogluconolactonase [Source:MGI Symbol;Acc:MGI:1913421]
CBFB	Cbfb	8	106	core binding factor beta [Source:MGI Symbol;Acc:MGI:99851]
DPEP2	Dpep2	8	107	dipeptidase 2 [Source:MGI Symbol;Acc:MGI:2442042]
APRT	Aprt	8	123	adenine phosphoribosyl transferase [Source:MGI Symbol;Acc:MGI:88061]
UBL5	Ubl5	9	21	ubiquitin-like 5 [Source:MGI Symbol;Acc:MGI:1913427]
DLAT	Dlat	9	51	dihydrolipoamide S-acetyltransferase (E2 component of pyruvate dehydrogenase complex) [Source:MGI Symbol;Acc:MGI:2385311]
ACAT1	Acat1	9	53	acetyl-Coenzyme A acetyltransferase 1 [Source:MGI Symbol;Acc:MGI:87870]

IDH3A	Idh3a	9	54	isocitrate dehydrogenase 3 (NAD+) alpha [Source:MGI Symbol;Acc:MGI:1915084]
ETFA	Etfa	9	55	electron transferring flavoprotein, alpha polypeptide [Source:MGI Symbol;Acc:MGI:106092]
HEXA	Hexa	9	59	hexosaminidase A [Source:MGI Symbol;Acc:MGI:96073]
CAMKV	Camkv	9	108	CaM kinase-like vesicle-associated [Source:MGI Symbol;Acc:MGI:2384296]
ARHGAP18	Arhgap18	10	27	Rho GTPase activating protein 18 [Source:MGI Symbol;Acc:MGI:1921160]
PDXK	Pdxk	10	78	pyridoxal (pyridoxine, vitamin B6) kinase [Source:MGI Symbol;Acc:MGI:1351869]
ATP5F1D	Atp5d	10	80	ATP synthase, H+ transporting, mitochondrial F1 complex, delta subunit [Source:MGI Symbol;Acc:MGI:1913293]
GNS	Gns	10	121	glucosamine (N-acetyl)-6-sulfatase [Source:MGI Symbol;Acc:MGI:1922862]
ATP5F1B	Atp5b	10	128	ATP synthase, H+ transporting mitochondrial F1 complex, beta subunit [Source:MGI Symbol;Acc:MGI:107801]
OGDH	Ogdh	11	6	oxoglutarate (alpha-ketoglutarate) dehydrogenase (lipoamide) [Source:MGI Symbol;Acc:MGI:1098267]
UQCRQ	Uqcrq	11	53	ubiquinol-cytochrome c reductase, complex III subunit VII [Source:MGI Symbol;Acc:MGI:107807]
PDLIM4	Pdlim4	11	54	PDZ and LIM domain 4 [Source:MGI Symbol;Acc:MGI:1353470]
ACADVL	Acadvl	11	70	acyl-Coenzyme A dehydrogenase, very long chain [Source:MGI Symbol;Acc:MGI:895149]
YWHAE	Ywhae	11	76	tyrosine 3-monooxygenase/tryptophan 5-monooxygenase activation protein, epsilon polypeptide [Source:MGI Symbol;Acc:MGI:894689]
CCL9	Ccl9	11	83	chemokine (C-C motif) ligand 9 [Source:MGI Symbol;Acc:MGI:104533]
SCPEP1	Scpep1	11	89	serine carboxypeptidase 1 [Source:MGI Symbol;Acc:MGI:1921867]
RPL38	Rpl38	11	115	ribosomal protein L38 [Source:MGI Symbol;Acc:MGI:1914921]
CD300LD3	Cd300ld3	11	115	CD300 molecule like family member D3 [Source:MGI Symbol;Acc:MGI:2687214]
ATP5PD	Atp5h	11	115	ATP synthase, H+ transporting, mitochondrial F0 complex, subunit D [Source:MGI Symbol;Acc:MGI:1918929]
RAB10	Rab10	12	3	RAB10, member RAS oncogene family [Source:MGI Symbol;Acc:MGI:105066]
RPL36A	Rpl36al	12	69	ribosomal protein L36A-like [Source:MGI Symbol;Acc:MGI:1913733]
RIOX1	Riox1	12	84	ribosomal oxygenase 1 [Source:MGI Symbol;Acc:MGI:1919202]
CDC42BPB	Cdc42bpb	12	111	CDC42 binding protein kinase beta [Source:MGI Symbol;Acc:MGI:2136459]
AKR1C13	Akr1c13	13	4	aldo-keto reductase family 1, member C13 [Source:MGI Symbol;Acc:MGI:1351662]

GMDS	Gmds	13	32	GDP-mannose 4, 6-dehydratase [Source:MGI Symbol;Acc:MGI:1891112]
UQCRB	Uqcrb	13	67	ubiquinol-cytochrome c reductase binding protein [Source:MGI Symbol;Acc:MGI:1914780]
GFM2	Gfm2	13	97	G elongation factor, mitochondrial 2 [Source:MGI Symbol;Acc:MGI:2444783]
MCCC2	Mccc2	13	100	methylcrotonoyl-Coenzyme A carboxylase 2 (beta) [Source:MGI Symbol;Acc:MGI:1925288]
STAB1	Stab1	14	31	stabilin 1 [Source:MGI Symbol;Acc:MGI:2178742]
LGALS3	Lgals3	14	48	lectin, galactose binding, soluble 3 [Source:MGI Symbol;Acc:MGI:96778]
CCAR2	Ccar2	14	70	cell cycle activator and apoptosis regulator 2 [Source:MGI Symbol;Acc:MGI:2444228]
DOK2	Dok2	14	71	docking protein 2 [Source:MGI Symbol;Acc:MGI:1332623]
DAB2	Dab2	15	6	disabled 2, mitogen-responsive phosphoprotein [Source:MGI Symbol;Acc:MGI:109175]
MTSS1	Mtss1	15	59	MTSS I-BAR domain containing 1 [Source:MGI Symbol;Acc:MGI:2384818]
EXOSC4	Exosc4	15	76	exosome component 4 [Source:MGI Symbol;Acc:MGI:1923576]
ACO2	Aco2	15	82	aconitase 2, mitochondrial [Source:MGI Symbol;Acc:MGI:87880]
RPL35A	Rpl35a	16	33	ribosomal protein L35A [Source:MGI Symbol;Acc:MGI:1928894]
ATP5PF	Atp5j	16	85	ATP synthase, H+ transporting, mitochondrial F0 complex, subunit F [Source:MGI Symbol;Acc:MGI:107777]
ATP5PO	Atp5o	16	92	ATP synthase, H+ transporting, mitochondrial F1 complex, O subunit [Source:MGI Symbol;Acc:MGI:106341]
NDUFB10	Ndufb10	17	25	NADH:ubiquinone oxidoreductase subunit B10 [Source:MGI Symbol;Acc:MGI:1915592]
HNRNPM	Hnrnpm	17	34	heterogeneous nuclear ribonucleoprotein M [Source:MGI Symbol;Acc:MGI:1926465]
KLC4	Klc4	17	47	kinesin light chain 4 [Source:MGI Symbol;Acc:MGI:1922014]
COMMD10	Commd10	18	47	COMM domain containing 10 [Source:MGI Symbol;Acc:MGI:1916706]
ACAA2	Acaa2	18	75	acetyl-Coenzyme A acyltransferase 2 (mitochondrial 3-oxoacyl-Coenzyme A thiolase) [Source:MGI Symbol;Acc:MGI:1098623]
NDUFS8	Ndufs8	19	4	NADH:ubiquinone oxidoreductase core subunit S8 [Source:MGI Symbol;Acc:MGI:2385079]
RBM14	Rbm14	19	5	RNA binding motif protein 14 [Source:MGI Symbol;Acc:MGI:1929092]
MPEG1	Mpeg1	19	12	macrophage expressed gene 1 [Source:MGI Symbol;Acc:MGI:1333743]
ANXA1	Anxa1	19	20	annexin A1 [Source:MGI Symbol;Acc:MGI:96819]
LIPA	Lipa	19	34	lysosomal acid lipase A [Source:MGI Symbol;Acc:MGI:96789]
PRDX3	Prdx3	19	61	peroxiredoxin 3 [Source:MGI Symbol;Acc:MGI:88034]

ACOT9	Acot9	X	154	acyl-CoA thioesterase 9 [Source:MGI Symbol;Acc:MGI:1928939]

*, Chromosome; **, Position (Mbp)
